# Diversity and mycotoxin production by *Fusarium temperatum* and *Fusarium subglutinans* as causal agents of pre-harvest *Fusarium* maize ear rot in Poland

**DOI:** 10.1007/s13353-018-0478-x

**Published:** 2018-11-15

**Authors:** Ł. Stępień, K. Gromadzka, J. Chełkowski, A. Basińska-Barczak, J. Lalak-Kańczugowska

**Affiliations:** 1 Institute of Plant Genetics, Polish Academy of Sciences, Poznań, ul. Strzeszyńska 34, 60-479 Poznań, Poland; 2 Department of Chemistry, Poznan University of Life Sciences, ul. Wojska Polskiego 75, 60-625 Poznań, Poland

**Keywords:** *Fusarium* species, Beauvericin, Enniatins, Maize ear rot, Translation elongation factor *tef*-1α

## Abstract

Maize ear rot is a common disease found worldwide, caused by several toxigenic *Fusarium* species. Maize ears and kernels infected by *Fusarium subglutinans* contained significant amounts of beauvericin, fusaproliferin, moniliformin, and enniatins. In 2011, *F*. *subglutinans* sensu lato has been divided into two species: *Fusarium temperatum* sp. nov. and *F*. *subglutinans* sensu stricto, showing different phylogeny and beauvericin production within the populations of maize pathogens in Belgium. Isolates of the new species—*F*. *temperatum*—were also identified and characterized in Spain, Argentina, Poland, France, and China as one of the most important pathogens of maize. Moreover, *F*. *temperatum* was proved to be pathogenic to maize seedlings and stalks. We identified *Fusarium* isolates obtained from diseased maize ears collected between 2013 and 2016 in Poland (321 isolates). Based on morphological analyses, six *Fusarium* species were identified. Molecular identification performed on the set of selected isolates (42 isolates) revealed 34 isolates to be *F*. *temperatum* and only five to be *F*. *subglutinans.* Interestingly, the phylogenetic analysis showed that the population of *F*. *temperatum* infecting maize in Poland remained quite uniform for over 30 years with only a few exceptions. For the first time, a single isolate of *Fusarium ramigenum* was detected from the area of Poland. Significant amounts of BEA were found in *Fusarium*-damaged kernels. The same kernel samples contained also enniatins A1, A, B1, and B. The results clearly demonstrate the occurrence of *F*. *temperatum* as maize pathogen in Poland for over the last three decades.

## Introduction

Maize ear rot is a common disease found worldwide, caused by several toxigenic *Fusarium* species and leading to the accumulation of several mycotoxins in kernels. The prevailing pathogenic species can vary over the years, depending on various factors such as the continent and region, agro-ecological conditions (Bottalico and Perrone 2002), and insect damage (Lew et al. 1991). Disease severity can also be influenced by other stress factors and susceptibility of cultivars (hybrids) to the infection by *Fusarium* species and to the accumulation of mycotoxins in kernels (Pascale et al. 2002).

The research on toxigenic fungal species and mycotoxins contaminating maize kernels has been conducted in Poland since 1984 in 15 growing seasons. *F*. *subglutinans* sensu lato was estimated as the prevailing species from 1985 until 1991, and it was accompanied by seven other species. Since 1995, the frequency of *Fusarium verticillioides* increased in most years, and this species outcompeted *F*. *subglutinans*, which frequency decreased, particularly in 1993 and 2006 seasons (Gromadzka et al. 2016). Mycotoxin moniliformin was the first toxin identified in the cultures of *F*. *subglutinans* isolates and in infected maize kernels from Poland (Sharman et al. 1991; Lew et al. 1996). Maize ears and kernels infected by *F*. *subglutinans* contained significant amounts of beauvericin (BEA), fusaproliferin (FP), moniliformin (MON), and enniatins (ENNs) (Logrieco et al. 1998; Gromadzka et al. 2016). MON has been shown to be toxic to animals and plants, whereas BEA and FP to insects, human cell lines, and chicken embryos and they play important roles in plant and animal diseases. BEA is entomopathogenic and highly toxic to insects (Logrieco et al.1998).

*F*. *subglutinans* is a member of the *Fusarium fujikuroi* species complex (FFSC) mating population E, known for many years as cosmopolitan species in agricultural and global environments, particularly being a pathogen of maize. The species was described for the first time by Nelson et al. (1983) in their manual, but at that time, no morphologically distinct subspecies could be found (Leslie and Summerell 2006). The number of recognized species of FFSC increased after over 15 years of DNA assays application and now over 50 distinct species are recognized (Aoki et al. 2014).

Isolates originating from Europe produced several mycotoxins: e.g., moniliformin, beauvericin, and fusaproliferin, both under laboratory and field conditions (Sharman et al. 1991; Logrieco et al. 1998; Kostecki et al. 1995, 1999; Krska et al. 1996; Lew et al. 1996).

Steenkamp et al. (2002) found two groups of *F. subglutinans* in a South African population of the species, showing distinct PCR-RFLP patterns. Additionally, isolates belonging to group 1 produced beauvericin (BEA) and isolates from group 2 were non-producers of BEA.

Moretti et al. (2008) examined the collection of 87 European *F*. *subglutinans* isolates using RFLP method and found two cryptic subspecies in these populations, which originated from Poland, Germany, Slovakia, Portugal, Italy, and former Yugoslavia. BEA was produced by 77% of isolates in amounts of 10–532 μg/g in rice cultures under laboratory conditions. Scauflaire et al. (2011) distinguished *F*. *temperatum* Scaufl. & Munaut species from *F*. *subglutinans* sensu lato within FFSC using the translation elongation factor gene (*tef-*1α) and *β*-tubulin (*tub-2*) DNA sequence analyses, mating compatibility and metabolite profiling, and described the species formally.

Fumero et al. (2015) isolated both *F*. *temperatum* and *F*. *subglutinans* from maize in Argentina. *F*. *temperatum* strains were BEA and fusaproliferin producers. All *F*. *subglutinans* isolates were fusaproliferin producers but none produced BEA.

Furthermore, both species were found in Spain, Poland, and France (Pintos et al. 2013; Czembor et al. 2014, 2015; Boutigny et al. 2017), and *F*. *temperatum* was proved to be pathogenic to maize seedlings and stalks. Wang et al. (2014) isolated *F*. *temperatum* from maize in China in 2009 and suggested that species produced fumonisins in infected ears under field conditions.

The aim of the present paper was to re-examine the isolates of *F*. *subglutinans* sensu lato from maize maintained 1984–1990 in the KF collection of pathogenic fungi at the Institute of Plant Genetics, Polish Academy of Sciences, Poznań, Poland (7 isolates), and to compare their identity and diversity to the 42 new isolates collected between 2013 and 2016 growing seasons using molecular analyses. Until recently, the balance between the frequencies of both species (*F*. *subglutinans* sensu stricto and *F*. *temperatum*) was not clear. Therefore, we tried to get a deep insight in the Polish population of the species and confront the data with the historical results. Additionally, *Fusarium* mycotoxin accumulation was examined in maize kernels.

## Materials and methods

### Fungal isolation and identification

Maize ear samples were collected in October 2013 (80 maize ear samples), 2014 (100 maize ear samples), 2015 (83 maize ear samples), and 2016 (58 maize ear samples) from maize fields in main maize-growing areas in Poland. Ears with significant ear rot symptoms were scored for the *Fusarium* ear rot rating (1–100% for moldy, discolored, and shrunken kernels), placed in separate paper bags, transported to the laboratory, and dried at room temperature. Then, surface mycelium and small pieces of kernels from each ear were placed in duplicate on agar plates with a low nutrient SNA medium to identify *Fusarium* species (Kostecki et al. 1995; Kwaśna et al. 1991). A tip of hyphae from each culture was transferred to both potato dextrose agar and synthetic SNA low nutrient agar. *Fusarium* species were identified according to Kwaśna et al. (1991) and Leslie and Summerell (2006).

*Fusarium* species were identified in all collected maize ears (321 samples) between 2013 and 2016. Based on this morphological identification, Table 1 was prepared. Then, *F*. *subglutinans* sensu lato isolates were selected for further molecular studies (2013, 1 isolate; 2014, 10 isolates; 2015, 20 isolates; 2016, 11 isolates). Additionally, we re-examined the isolates of *F*. *subglutinans* sensu lato from maize, maintained in the culture collection and isolated between 1984 and 1990 (7 isolates).

**Table 1:** *Fusarium* species isolated from maize with ear rot symptoms in 2013–2016 seasons in Poland based on morphological identification

Year	Frequency of *Fusarium* species [%]					
	*F*. *cul*	*F*. *prolif*	*F*. *gram*	*F*. *poae*	*F*. *sub*	*F*. *vert*
2013*	0	0	7.1	45.7	3.1	44.1
2014*	0	0	13.2	14.0	26.3	46.5
2015	2.7	4.0	5.3	20.0	28.0	40.0
2016	0	4.5	7.2	26.0	27.6	34.7

*According to Gromadzka et al. (2016)

*F*. *prolif*, *Fusarium proliferatum* (Matsushima) Nirenberg; *F*. *cul*, *Fusarium culmorum* (W.G. Smith) Saccardo; *F*. *gram*, *Fusarium graminearum* Schwabe; *F*. *poae*, *Fusarium poae* (Peck) Wollenw; *F*. *sub*, *Fusarium subglutinans* sensu lato (Wollenw. & Reinking) Nelson, Toussoun & Marasas; *F. vert*, *Fusarium verticillioides* (Saccardo) Nirenberg (*F*. *moniliforme* Sheldon)

New isolates from harvest seasons 2013–2014 (accessions with “K” designation) and isolates originating from maize kernels from harvest seasons 1984–1990, deposited in the culture collection of the Institute of Plant Genetics, Poznań, Poland (accessions with “KF” designation) and the Institute of Sciences of Food Production, CNR, Bari, Italy (accessions with “ITEM” designation), were used to examine their genetic diversity based on partial *tef-*1α sequence analysis.

Fungal isolates were stored on SNA slants in the refrigerator and in sterile 18% glycerol at − 75 °C.

The frequency of *F*. *subglutinans* occurrence (1985–2014) was based partly on previous studies (Gromadzka et al. 2016) and partly on present studies (2015–2016) for comparison.

### Molecular and phylogenetic analyses

Growing mycelia of individual fungal isolates were maintained in pure cultures for 7 days on PDA medium. Genomic DNA was extracted using a modified CTAB (hexadecyltrimethylammonium bromide) (method described earlier by Stępień et al. (2013)). The DNA extracts were stored at − 20 °C. Species identification was done on the basis of the sequences of the translation elongation factor 1 alpha (*tef-*1α). Polymerase chain reactions were performed as described earlier by Stępień et al. (2013), using DreamTaq Green DNA polymerase (Thermo Scientific, Espoo, Finland). Amplicons of ca. 600 bp in length were electrophoresed in 1.5% agarose gel (Invitrogen) with GelGreen Nucleic Acid Stain (Biotium, Inc.)*.* For sequence analysis, PCR-amplified DNA fragments were labeled using forward primer and the BigDyeTerminator 3.1 kit (Applied Biosystems, Foster City, CA, USA), according to producer’s recommendations, and precipitated with 96% ethanol. Sequence reading was performed using Applied Biosystems equipment. Sequences were analyzed using BLASTn algorithm. The dendrogram was calculated using MEGA 4 software package (Tamura et al. 2007) using *F*. *oxysporum* (GenBank sequence) and *F*. *nygamai* KF 337 (home made sequence) as an outgroup. Maximum parsimony heuristics were used with “fill gaps” function. Bootstrapping was done using 1000 iterations. Only branches with at least 50% of support were considered and presented.

### Mycotoxin analyses

Mycotoxins were analyzed in maize ear samples, which were collected in the years 2013–2016. Kernels of ears were manually separated into two fractions: Fusarium-damaged kernels (FDK) and healthy-looking kernels (HLK—symptomless kernels). Then kernels of the FDK fraction of samples naturally colonized by *F*. *subglutinans* sensu lato were subjected to chemical analyses of mycotoxins using methodologies described below.

In the case of maize ears number K116, K308.1, K308.2, K343, K419, and K429, the amounts of the FDK fractions were insufficient to determine mycotoxins.

#### Chemicals and reagents

Mycotoxin standards (MON; Enniatins: A, A1, B, and B1; BEA) and all chemicals were supplied by Sigma-Aldrich (Steinheim, Germany). Water of HPLC grade from our own Millipore water purification system was used for analyses.

#### Sample preparation, extraction, and HPLC analysis

Mycotoxin content was determined using the chromatographic system: a Waters 2695 high-performance liquid chromatograph, a Waters 2475 Multi λ Fluorescence Detector, and/or a Waters 2996 Array Detector.

Enniatins and beauvericin were identified and quantified as reported by Jestoi et al. (2004). The MON content was analyzed according to the method described by Kostecki et al. (1999).

## Results and discussion

### Diversity of the *Fusarium* isolates

**Fig. 1:**
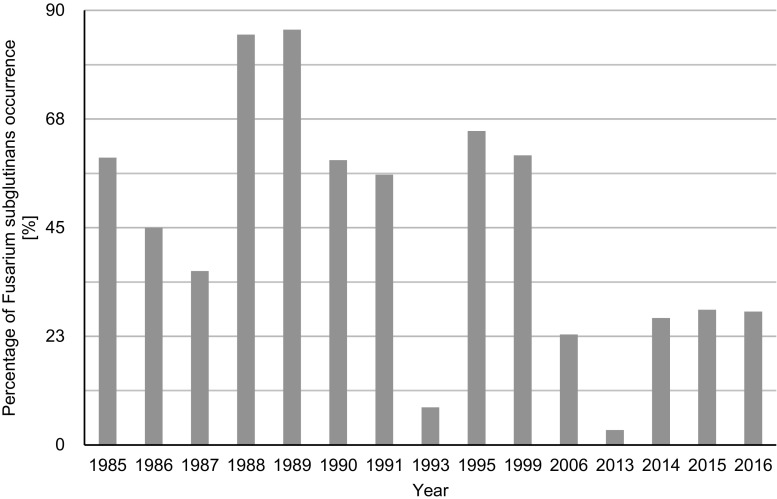
Percentage of *Fusarium subglutinans* sensu lato (*F*. *subglutinans* sensu stricto and *F*. *temperatum*) occurrence [%] in Poland in the years 1985–2016

Among all isolates obtained during the 4-year study, at least six species were identified based on morphological analyses (Table 1). Three *Fusarium* species were identified in moldy maize ears in two localities studied with the high frequencies: *F*. *verticillioides* (34.7–46.5%), *F*. *subglutinans* sensu lato (3.1–28.0%), and *F*. *poae* (14.0–45.7%). *F*. *subglutinans* sensu lato was found in maize ear samples on the area of Poland in 15 seasons since 1984 until 2016, and the species frequencies were ranging from 3.1 up to 86% of the total *Fusarium* isolates (Fig. 1).

Frequency of *F*. *subglutinans* sensu lato was particularly high in harvest years 1985–1991 and also in 1995 and 1999 seasons. High content of moniliformin up to 4 mg/kg was found in the fraction of *F*. *subglutinans*–damaged kernels of those samples (Lew et al. 1996). In the last year, the frequency of *F*. *subglutinans* sensu lato increased (3.1–28.0%) with the decreasing frequency of *F*. *verticillioides* (44.1–34.7%). *F*. *proliferatum* was found in 2015 and 2016 seasons with the frequencies of 4.0 and 4.5%, respectively (Table 1). Considering *F*. *subglutinans* and *F*. *verticillioides*, their frequencies significantly changed (Gromadzka et al. 2016). In the years 1988–1990, the discussed species were at the level of 85–56% and 3%, respectively.

Scauflaire et al. (2011) have divided *F*. *subglutinans* sensu lato into the two species, *F*. *temperatum* sp. nov. and *F*. *subglutinans*, showing different phylogeny and beauvericin production within the populations of maize pathogens in Belgium. Isolates of the new species—*F*. *temperatum*—were also identified and characterized in Spain (Pintos et al. 2013), Argentina (Fumero et al. 2015), Poland (Czembor et al. 2014, 2015), France (Boutigny et al. 2017), and China (Wang et al. 2014) among the most important pathogens of maize.

That is why we decided to check how the population of these two species is shaped in Poland and which of the two discussed species dominates nowadays compared to the 1985–1999 period.

**Fig. 2:**
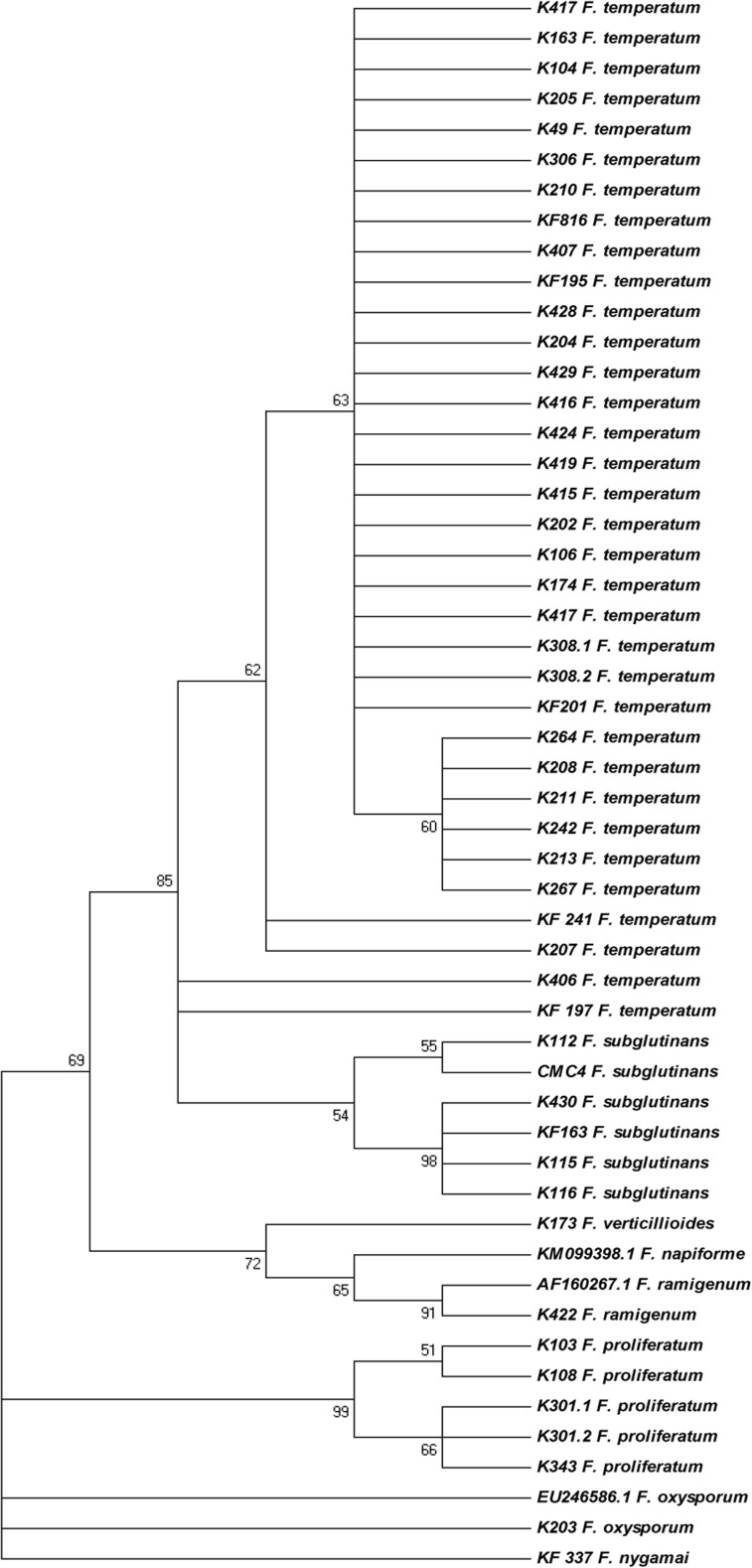
Maximum parsimony dendrogram of *Fusarium temperatum* and *F*. *subglutinans* isolates obtained during 2013–2016 seasons from maize ear diseased samples from two localities in Poland. Additionally, KF collection isolates were included, along with some other species for reference (*F*. *proliferatum*, *F*. *verticillioides*, *F*. *oxysporum*, and *F*. *ramigenum*)

Moretti et al. (2008) examined 43 isolates of *F*. *subglutinans* sensu lato originating from maize in Poland from harvest seasons between 1984 and 1990. Using RFLP method and testing their ability to produce BEA, two groups of isolates were distinguished: 34 were proven to be BEA and fusaproliferin producers belonging to the group 1 (and corresponding to *F*. *temperatum*, according to Scauflaire et al. 2011). Three isolates were BEA non-producers and belonged to the group 2, corresponding to *F*. *subglutinans* sensu stricto. Those results were confirmed in our study by the identification of 34 isolates (2013–2016) as representing group 1, actually to be *F*. *temperatum* (Fig. 2, Table 2)*.* Additionally, five isolates from our collection (KF 816, KF 1955, KF 197, KF 201, and KF 241), which were previously described as *F*. *subglutinans* by Kostecki et al. (1995, 1999), were confirmed to be *F*. *temperatum* using the analysis of the *tef*-1α gene sequence (Table 2). Only one isolate corresponded to *F*. *subglutinans* sensu stricto (KF 163).

**Table 2:** *Fusarium subglutinans*, *F*. *temperatum*, and *F*. *proliferatum* isolates analyzed in this study

Year of isolation	Origin	Isolate code and number	Species identified using *tef*-1α gene sequence analysis
1984	Poland	KF 816 nt	*F*. *temperatum*
1984	Poland	KF 163 (ITEM 1348) f, a	*F*. *subglutinans*
1984	Poland	KF 195 (ITEM 1349) b, f, a	*F*. *temperatum*
1984	Radzików, Poland	KF 197 (ITEM 1422) b, f, a	*F*. *temperatum*
1984	Poland	KF 201 (ITEM 1352) b, f, a	*F*. *temperatum*
1984	Poland	KF 241 (ITEM 1353) f, a	*F*. *temperatum*
Unknown	USA	KF 337 (NRRL 25312) c	*F*. *nygamai*
2013	Złotniki, Poland	K 49 bn	*F*. *temperatum*
2014	Złotniki, Poland	K 103 bn	*F*. *proliferatum*
2014	Złotniki, Poland	K 104 bn	*F*. *temperatum*
2014	Złotniki, Poland	K 106 bn	*F*. *temperatum*
2014	Złotniki, Poland	K 108 bn	*F*. *proliferatum*
2014	Złotniki, Poland	K 112 bn	*F*. *subglutinans*
2014	Złotniki, Poland	K 115 bn, b	*F*. *subglutinans*
2014	Złotniki, Poland	K 116 bn, b	*F*. *subglutinans*
2014	Kobierzyce, Poland	K 163 bn	*F*. *temperatum*
2014	Kobierzyce, Poland	K 173 bn	*F*. *verticillioides*
2014	Kobierzyce, Poland	K 174 bn	*F*. *temperatum*
2015	Swadzim, Poland	K 201 bn	*F*. *temperatum*
2015	Swadzim, Poland	K 202 bn	*F*. *temperatum*
2015	Swadzim, Poland	K 203 bn	*F*. *oxysporum*
2015	Swadzim, Poland	K 204 bn	*F*. *temperatum*
2015	Swadzim, Poland	K 205 bn	*F*. *temperatum*
2015	Swadzim, Poland	K 207 bn	*F*. *temperatum*
2015	Swadzim, Poland	K 208 bn	*F*. *temperatum*
2015	Swadzim, Poland	K 210 bn	*F*. *temperatum*
2015	Swadzim, Poland	K 211 bn	*F*. *temperatum*
2015	Swadzim, Poland	K 213 bn	*F*. *temperatum*
2015	Kobierzyce, Poland	K 242 bn	*F*. *temperatum*
2015	Kobierzyce, Poland	K 264 bn	*F*. *temperatum*
2015	Kobierzyce, Poland	K 267 bn	*F*. *temperatum*
2015	Kobierzyce, Poland	K 270 bn	*F*. *proliferatum*
2015	Kwieciszewo, Poland	K 301.1 bn	*F*. *proliferatum*
2015	Kwieciszewo, Poland	K 301.2 bn	*F*. *proliferatum*
2015	Kwieciszewo, Poland	K 306 bn	*F*. *temperatum*
2015	Kwieciszewo, Poland	K 308.1 bn	*F*. *temperatum*
2015	Kwieciszewo, Poland	K 308.2 bn	*F*. *temperatum*
2015	Komalwy, Poland	K 343 bn	*F*. *proliferatum*
2016	Złotniki, Poland	K 406 bn	*F*. *temperatum*
2016	Złotniki, Poland	K 407 bn	*F*. *temperatum*
2016	Złotniki, Poland	K 415 bn	*F*. *temperatum*
2016	Złotniki, Poland	K 416 bn	*F*. *temperatum*
2016	Złotniki, Poland	K 417 bn	*F*. *temperatum*
2016	Złotniki, Poland	K 419 bn	*F*. *temperatum*
2016	Złotniki, Poland	K 424 bn	*F*. *temperatum*
2016	Złotniki, Poland	K 428 bn	*F*. *temperatum*
2016	Złotniki, Poland	K 429 bn	*F*. *temperatum*
2016	Złotniki, Poland	K 430 bn	*F*. *subglutinans*
2016	Złotniki, Poland	K 422 bn	*F*. *ramigenum*

a - according to Logrieco et al. (1996)

b - isolates proved to be BEA producers under laboratory conditions (Kostecki et al. 1995)

c - according to Azor et al. (2009)

bn - BEA present in ears exhibiting *Fusarium* ear rot under field conditions

f - isolates proved to be fusaproliferin producers (Kostecki et al. 1999)

Interestingly, the phylogenetic analysis showed that the population of *F*. *temperatum* infecting maize in Poland remains quite uniform (Fig. 2), since most of the collection strains and newly isolated genotypes of the species grouped on the same branch, which means that the sequences were identical. There were only few exceptions that were genetically distinct from that group: K406 and KF 197, KF 241, and K207 grouped outside of the main group. There was also a small group of isolates very similar to each other: K264, K208, K211, K242, K213, and K267. It can be concluded that there are no significant differences between the *F*. *temperatum* populations present in Poland some 20 years ago and those present now. This may suggest that the population propagates clonally with the maize seed material. However, it is not possible to draw a similar conclusion regarding *F*. *subglutinans* sensu stricto, since there were too few isolates to be analyzed. Detailed analysis of this species requires additional strain isolation and studies. Interestingly, detailed molecular identification procedure allowed to detect a single isolate of *F*. *ramigenum*. To our best knowledge, this is the first time this species was identified in Poland. The strain K270 of *F*. *proliferatum* gave the sequence read of poor quality; therefore, we decided to exclude it from the phylogenetic analysis.

To improve clarity and put emphasis on the *F*. *subglutinans* and *F*. *temperatum* species, only one *F*. *verticillioides* isolate was included in the phylogenetic analysis for reference, along with single isolates of *F*. *oxysporum* and *F*. *ramigenum* and five isolates of *F*. *proliferatum* (Fig. 2).

The frequency of *F*. *temperatum* was significantly higher than that of *F*. *subglutinans* in Poland and in other countries as well. The frequency of *F*. *proliferatum* increased in 2015 and 2016 seasons, comparing to the earlier years. This finding may be particularly important because of high toxigenicity of *F*. *proliferatum*, producing the same mycotoxins as *F*. *temperatum* but additionally fumonisins. The spread of *Fusarium* species caused recently increase in frequencies of species such as *F*. *graminearum* and *F*. *verticillioides* in other countries of moderate climate, including Poland and Austria (Adler et al. 2002; Gromadzka et al. 2016).

The description of *F*. *subglutinans* isolates found in manuals available before 2011 was not detailed enough to distinguish *F*. *subglutinans* sensu stricto from *F*. *temperatum* using morphological characters only (Nelson et al. 1983; Leslie and Summerell 2006). Implementing molecular identification, in particular, the analysis of the sequence of *tef*-1α gene, provided a method to identify closely related species such as about 15 members of FFSC species complex (Stępień et al. 2011, 2013; Aoki et al. 2014). According to Aoki et al. (2014), FFSC comprises of 13 species that are known to reproduce sexually and are heterothallic. On the other hand, molecular phylogenetic studies have revealed that FFSC is represented by over 50 phylogenetically distinct species. Over 300 species of *Fusarium* have been recognized using DNA assays; however, fewer than a half of this number have been formally described. Continuous changes in *Fusarium* species identification and nomenclature require careful edition of new modern reviews and manuals for verification and dissemination of this knowledge. *F*. *temperatum* Scaufl. & Munaut has not yet been included in the manuals on *Fusarium* taxonomy; however, it was already accepted in several scientific papers mentioned above.

### Mycotoxins production by *F*. *temperatum* and *F*. *subglutinans* sensu stricto

Of all the known mycotoxins, beauvericin, moniliformin, and enniatins were accumulated in maize kernels infected by *F*. *temperatum*.

Beauvericin production under laboratory conditions on solid substrate (polished rice) is often helpful in confirming the identification of species (Kostecki et al. 1999; Fumero et al. 2015). Until now, only few isolates of *F*. *temperatum* were studied in detail concerning their potential to synthesize BEA and enniatins in vitro (Stępień and Waśkiewicz 2013). One of them produced only BEA, one BEA and all four enniatin analogs measured (KF 3321 from pineapple), and some produced mixtures of BEA and some enniatins but not all. Therefore, the production of other mycotoxins (such as enniatins) by *F*. *temperatum* isolates should be examined in future for a larger number of isolates to understand the profiles of these mycotoxins for *F*. *temperatum* and *F*. *subglutinans* fully.

BEA and MON were produced by 19 isolates of *F*. *subglutinans* sensu lato examined in Poland in 1993 and, under laboratory conditions, the efficiencies ranged between 201 and 4000 mg/kg for BEA and 0.9–381 mg/kg for MON. The strain KF 534 (ITEM 1434)—considered to be the model strain—was found to produce the highest amounts of MON (4 g/kg) and also FP and BEA on polished rice under laboratory conditions (Kostecki et al. 1995, 1999) This strain was confirmed to be *F*. *subglutinans* group 1, later known to be *F*. *temperatum*.

Isolate KF 555 was previously found to produce high amounts of BEA in kernels of nine maize hybrids inoculated under field conditions (Krska et al. 1996). *Fusarium*-damaged kernels accumulated 6.58–15.22 mg/g of BEA, and the lowest amounts were accumulated by the hybrid “Anna,” which was found to be of the lowest susceptibility to infection and mycotoxin accumulation. Healthy-looking kernels from the same ears accumulated very low amounts of BEA (Krska et al. 1996).

Significant amounts and frequencies of BEA contamination were found in *Fusarium*-damaged kernels in our survey of field maize samples harvested in 2013–2016 seasons and colonized by *F*. *subglutinans* sensu lato (Table 3). The same kernel samples contained also enniatins A1, A, B1, and B.

**Table 3:** Moniliformin, beauvericin, and enniatins identified in kernels colonized with *Fusarium temperatum*, *F*. *subglutinans*, and *F*. *proliferatum* species under field conditions in 2013, 2014, 2015, and 2016 seasons (*nd*, not detected; *na*, not analyzed)

Species	Sample number	MON (μg/g)	BEA (μg/g)	Enniatins			
				B (μg/g)	B1 (μg/g)	A (μg/g)	A1 (μg/g)
*F*. *temperatum*	K49	nd	16.60	nd	nd	nd	47.82
*F*. *proliferatum*	K103	12.05	51.45	nd	nd	24.90	nd
*F*. *temperatum*	K104	7.84	71.17	nd	nd	nd	42.93
*F*. *temperatum*	K106	0.65	33.79	104.39	nd	nd	24.78
*F*. *proliferatum*	K108	8.80	34.07	58.98	nd	nd	34.49
*F*. *subglutinans*	K112	9.93	98.94	nd	nd	nd	16.89
*F*. *subglutinans*	K115	4.36	106.34	nd	nd	10.78	24.46
*F*. *subglutinans*	K116	na	na	na	na	na	na
*F*. *temperatum*	K163	13.52	91.75	nd	nd	nd	nd
*F*. *temperatum*	K174	118.48	nd	nd	nd	nd	nd
*F*. *temperatum*	K201	4.32	9.95	nd	14.08	14.86	nd
*F*. *temperatum*	K202	5.62	19.04	nd	nd	38.49	nd
*F*. *temperatum*	K204	3.69	48.31	nd	nd	17.08	nd
*F*. *temperatum*	K205	3.24	33.56	nd	13.92	9.44	nd
*F*. *temperatum*	K207	0.03	52.26	nd	29.67	11.23	nd
*F*. *temperatum*	K208	0.15	6.58	nd	7.48	16.28	nd
*F*. *temperatum*	K210	3.75	71.56	nd	26.79	13.53	5.55
*F*. *temperatum*	K211	0.02	49.56	nd	47.21	73.73	nd
*F*. *temperatum*	K213	nd	nd	nd	45.13	15.66	46.03
*F*. *temperatum*	K242	1.26	126.08	nd	8.36	39.14	nd
*F*. *temperatum*	K264	2.91	457.31	130.63	nd	46.51	325.35
*F*. *temperatum*	K267	0.03	392.72	109.68	8.61	48.90	7.66
*F*. *proliferatum*	K270	0.06	240.30	124.35	nd	50.97	44.71
*F*. *proliferatum*	K301	0.04	1.70	60.17	nd	nd	3883.28
*F*. *temperatum*	K306	0.03	7.50	42.02	nd	13.29	972.45
*F*. *temperatum*	K308.1	na	na	na	na	na	na
*F*. *temperatum*	K308.2	na	na	na	na	na	na
*F*. *proliferatum*	K343	na	na	na	na	na	na
*F*. *temperatum*	K406	90.53	64.33	nd	nd	38.43	nd
*F*. *temperatum*	K407	68.21	40.11	nd	nd	120.86	nd
*F*. *temperatum*	K415	14.26	151.69	nd	nd	29.78	nd
*F*. *temperatum*	K416	3.21	164.10	nd	nd	390.48	nd
*F*. *temperatum*	K417	3.30	48.70	nd	nd	80.19	nd
*F*. *temperatum*	K419	na	na	na	na	na	na
*F*. *temperatum*	K424	9.85	55.08	nd	36.84	111.59	nd
*F*. *temperatum*	K428	75.55	76.88	nd	nd	132.27	nd
*F*. *temperatum*	K429	na	na	na	na	na	na
*F*. *subglutinans*	K430	10.50	37.42	nd	nd	110.52	nd
*F*. *ramigenum*	K422	24.76	37.71	nd	nd	315.15	nd

Comparing the amounts of mycotoxins synthesized in maize kernels by *F*. *temperatum* and *F*. *subglutinans*, high differences were noticed, in particular regarding moniliformin and enniatin A1. In FDK fraction infected by *F*. *temperatum*, the average amount of MON (17.22 mg/kg) was almost twice as high as in kernels (8.26 mg/kg) from which *F*. *subglutinans* was identified. In the case of the enniatin A1, the differences were even higher and amounts of the toxin accumulated in kernels infected by *F*. *temperatum* and *F*. *subglutinans* were on the average at the levels of 58.90 mg/kg and 13.78 mg/kg, respectively. The analysis of mycotoxins also showed that in the case of *F*. *subglutinans*, no enniatins B and B1 were found, whereas, in the case of kernels infected by *F*. *temperatum*, they were on average at the level of 15.47 and 9.52 mg/kg, respectively. In addition, both species were characterized by a similar amount of synthesized beauvericin (about 80 mg/kg) as well as enniatin A (about 45 mg/kg).

Both in rice cultures and under field conditions, *F*. *temperatum* was found to be BEA, MON, and FP producer and contributed to the contamination of maize kernels with the abovementioned mycotoxins and also enniatins A1, A, B, and B1 (Tables 2 and 3).

The results clearly demonstrate the occurrence of *F*. *temperatum* as maize pathogen in Poland for over the last three decades (since 1984) and recent literature has reported the species also in several other countries mentioned above.
